# Immune checkpoint inhibitor induced bullous pemphigoid treated by dupilumab: A case series

**DOI:** 10.1016/j.jdcr.2024.11.033

**Published:** 2024-12-11

**Authors:** Emily Bogdanski, Matthew D. Viveiros, Catherine Chung, Brittany Dulmage

**Affiliations:** a The Ohio State University, College of Medicine, Columbus, Ohio; b Department of Dermatology, The Ohio State University Wexner Medical Center, Columbus, Ohio

**Keywords:** autoimmune, axitinib, bullous pemphigoid, dermatology, dupilumab, immune checkpoint inhibitor, ipilimab, nivolumab, pembrolizumab

## Introduction

While revolutionary in the treatment of various malignancies, immune checkpoint inhibitors (ICIs) are known to induce autoimmune bullous pemphigoid (ICI-BP). Often, ICI-BP manifests with a relatively more severe disease course than BP, characterized by prolonged pruritus refractory to treatment.^1^ Several case reports demonstrate that dupilumab provides relief and can induce remission from ICI-BP, though length of treatment required and ability to induce sustained resolution are unknown.^1^, ^2^, ^3^ The objective of this case series is to highlight all five patients who experienced improvement in their ICI-BP following treatment with 6 months or more of dupilumab in our tertiary care network (Table I).

**Table I:** Overview of five cases of immune checkpoint inhibitor induced bullous pemphigoid (ICI-BP)

	Patient				
	1	2	3	4	5
Demographics					
Age	69	86	68	74	73
Gender	M	F	M	M	M
Original diagnosis and treatment					
Diagnosis	Renal clear cell carcinoma	Invasive ductal carcinoma and soft tissue sarcoma	Melanoma	Squamous cell carcinoma of head and neck	Melanoma
Treatment (immune checkpoint inhibitor)	Pembrolizumab	Nivolumab, ipilimumab	Nivolumab	Pembrolizumab	Nivolumab
Treatment (non-immune checkpoint inhibitor)	Axitinib	Anastrozole, ribociclib	n/a	Carboplatin, paclitaxel	n/a
ICI-BP onset, biopsy, antibodies, and treatment					
Cycles until ICI-BP	15	27	13	7	10
ICI-BP biopsy key findings	H&E: subepidermal vesicle filled with eosinophils. DIF: linear IgG and C3 along the basement membrane and IgM within numerous cytoid bodies.	H&E: subepidermal blister with dermal inflammation containing eosinophils and neutrophils. DIF: linear IgG and C3 deposition along the basement membrane zone.	H&E: subepidermal clefting associated with a dermal perivascular lymphocytic infiltrate with numerous eosinophils. DIF: non-continuous basement membrane zone deposition of IgG and C3 and to a lesser extent C5b-9.	H&E: dermal inflammation containing eosinophils with marked eosinophilic spongiosis and eosinophils tagging along the dermoepidermal junction.	H&E: epidermal acanthosis and eosinophilic spongiosis with numerous eosinophils aligning the dermoepidermal junction; abundant eosinophils and scattered neutrophils in the dermis DIF: linear staining by C3 along the basement membrane zone.
ICI-BP antibodies	BP-180 positive	BP-180 positive	BP-180 and BP-230 negative	BP-180 and BP-230 positive	
Initial ICI-BP treatment (non-dupilumab)	Topical triamcinolone, topical clobetasol	Oral prednisone, topical triamcinolone	Topical fluocinonide, oral prednisone	Diphenhydramine, oral prednisone, topical hydrocortisone, topical triamcinolone	Topical hydrocortisone, topical triamcinolone, intramuscular kenalog, oral prednisone
Dupilumab length of treatment	651	270	790	262	539
Days without reappearance of BP	227	Treatment ongoing	128	n/a	469
Days until recurrence of BP	n/a	n/a	128	n/a	n/a
Initial recurring BP symptoms	None	None	Pruritic urticarial lesions/plaques	None	None
Living/Deceased	Living	Living	Living	Deceased	Living

*BP*, Bullous pemphigoid; *DIF*, direct immunofluorescence; *H&E*, hematoxylin and eosin; *ICI*, immune checkpoint inhibitor.

## Cases

### Case 1

A 69-year-old male who received 15 cycles of axitinib and pembrolizumab for metastatic renal clear cell carcinoma was diagnosed with ICI-BP following biopsy and positive BP180 antibodies. The patient’s immunotherapy was discontinued. Dupilumab was initiated after failing topical triamcinolone and clobetasol. At his 8-week follow-up, the patient reported improvement in his symptoms. The resolution of his BP was confirmed with negative antibodies 651 days following initiation of dupilumab and has remained without BP reappearance for 227 days.

### Case 2

An 86-year-old female who received nivolumab, ipilimumab, ribociclib, and anastrozole for metastatic invasive ductal carcinoma and soft tissue sarcoma was confirmed to have ICI-BP after 27 cycles with biopsy and positive BP180. She was trialed on oral steroids without symptomatic relief. Subsequently, dupilumab was trialed and has significantly improved her symptoms after 9 months of injections. She first noted improvement at her 3-week follow-up appointment. She continues on dupilumab therapy.

### Case 3

A 68-year-old male with metastatic melanoma was confirmed to have ICI-BP following biopsy after 13 cycles of nivolumab. Failed treatments included topical fluocinonide and oral prednisone before beginning dupilumab. The patient stated that he was experiencing minimal flares at his 2-month follow-up appointment. After 790 days, the patient achieved resolution of BP, confirmed by negative BP antigen antibodies. However, a pruritic rash developed 128 days after dupilumab discontinuation, suspicious for ICI-BP, and he was restarted on dupilumab.

### Case 4

A 74-year-old male with metastatic squamous cell carcinoma of the head and neck was treated with seven cycles of carboplatin, paclitaxel, and pembrolizumab before developing ICI-BP with elevated BP180 and BP230 antibodies. The patient continued his immunotherapy for another 2 months following the diagnosis of ICI-BP before discontinuation. He was started on oral steroids and diphenhydramine without significant relief. He began dupilumab injections but passed away before achieving resolution of his ICI-BP after 262 days of being on dupilumab. At the time of his 3-month follow-up appointment after starting dupilumab, the patient was still experiencing pruritus, though it was improved from prior to starting dupilumab.

### Case 5

**Fig 1:**
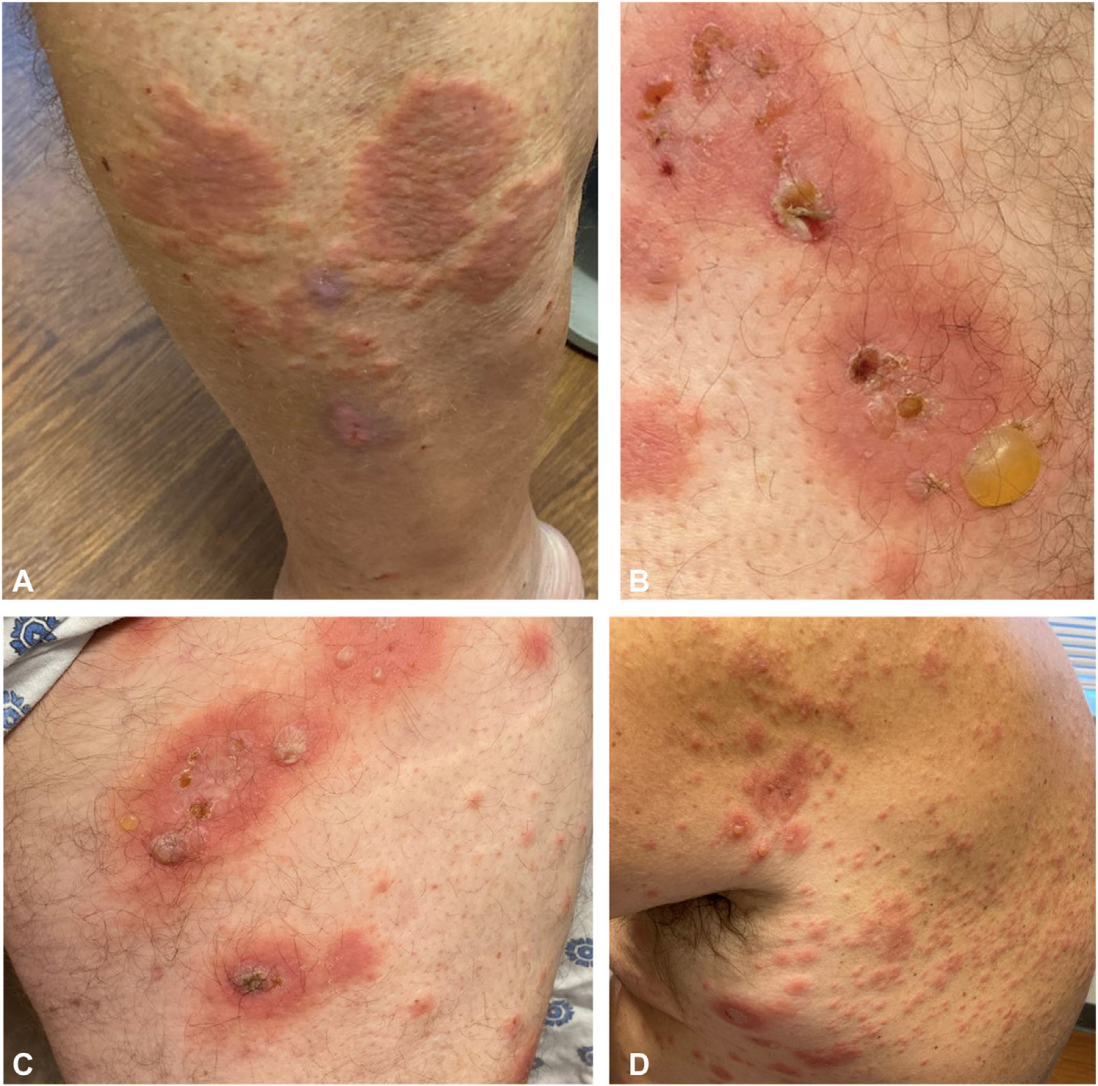
Photographs of ICI-BP seen in case 5 prior to starting dupilumab on the (**A**) calf, (**B**) thigh, (**C**) thigh, and (**D**) lateral back showing widespread urticarial plaques with overlying tense and ruptured bullae. *ICI-BP*, Immune checkpoint inhibitor induced bullous pemphigoid.

**Fig 2:**
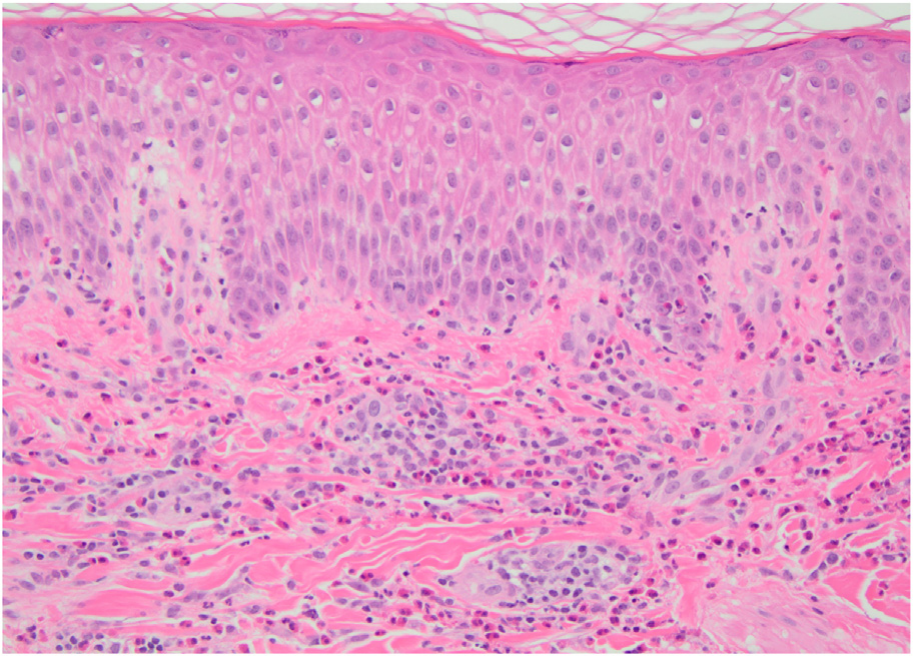
Skin biopsy demonstrating numerous eosinophils tagging the dermal-epidermal junction and in the upper dermis, consistent with an urticarial lesion of BP (hematoxylin and eosin, 200×). Direct immunofluorescence (not shown) demonstrated 3+ linear C3 and IgG basement membrane zone positivity. *BP*, Bullous pemphigoid.

**Fig 3:**
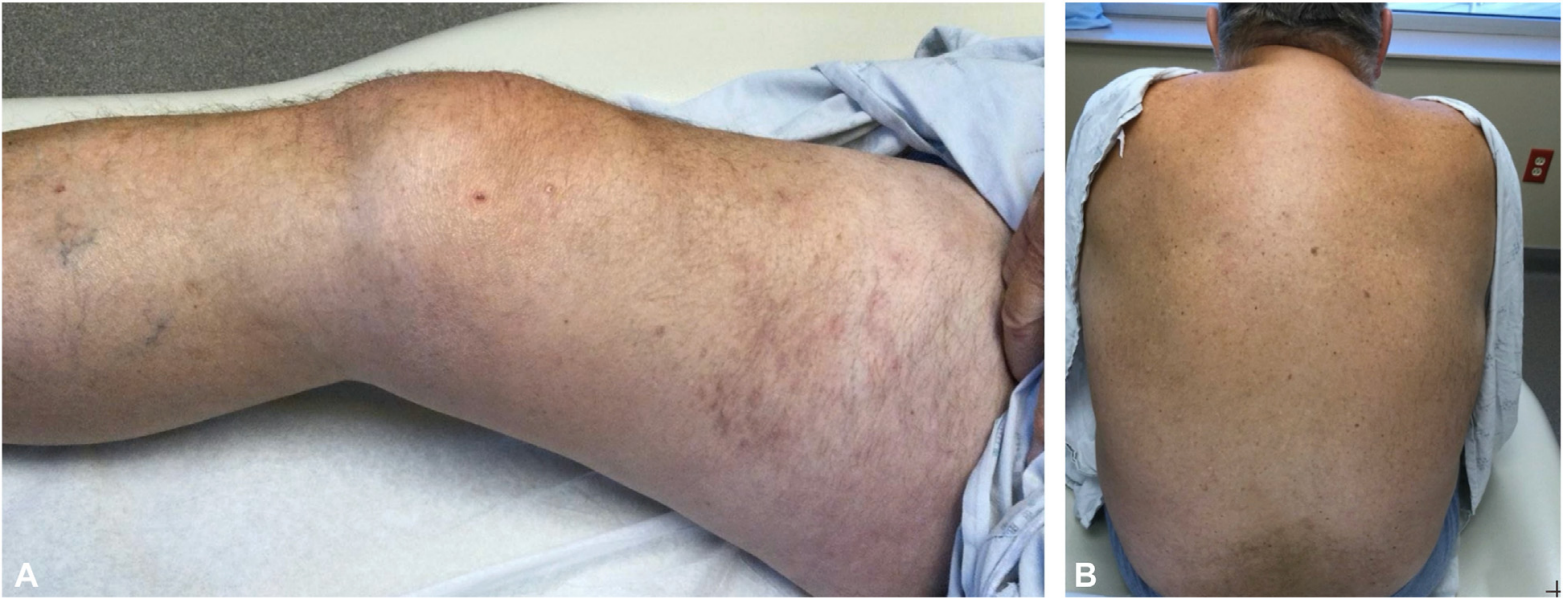
Improvement following dupilumab treatment in case 5 with total clearance of skin lesions on (**A**) thigh and (**B**) back.

A 73-year-old male with malignant melanoma developed ICI-BP after 10 cycles of nivolumab, confirmed by biopsy (Figs 1 and 2). The patient completed three additional cycles of nivolumab before being placed under surveillance. Initially, he was treated with oral and topical steroids without relief before beginning dupilumab. He continued dupilumab for 539 days before achieving remission of his BP (Fig 3), confirmed by negative antibodies. At his 6-week follow-up appointment, the patient reported improvement in his symptoms. He has remained without BP reappearance for 469 days.

## Discussion

Cutaneous toxicities are the most common immune-related adverse effect across ICIs due to activation of CD4+/CD8+ T-cells.^3^ In a multicenter observational study, ICI-bullous skin toxicity was seen in 22/7391 (0.3%) patients being treated with ICIs; the median time to onset of bullous adverse events was 12 months, and median symptom duration was 6 months.^4^ When considering treatment for recalcitrant ICI-BP, dupilumab—an IL-4/IL-13 blocker—is ideal as a steroid-sparing agent (SSA), foregoing immunosuppression. It is well-tolerated, limiting inflammation by decreasing T-helper 2 differentiation, IgE production, and eosinophil recruitment, and can be effectively combined with other immunotherapies.^3^ As a result of its efficacy in various BP/ICI-BP cohorts as an SSA, dupilumab is currently under investigation in phase 2/3 trials for BP treatment.^1^^,^^5^ Hence, dupilumab was chosen for these five patients.

All five patients achieved relief of their ICI-BP symptoms on dupilumab. Two of the five achieved full resolution, and one achieved full resolution for nearly 4 months before recurrence. The number of ICI cycles before BP onset varied between 7 and 27, with an average of 14.4 cycles prior to ICI-BP diagnosis. The length of dupilumab treatment ranged from 270 to 790 days, with an average of 502.4 days. Determining the time to clinical improvement is restricted by each patient’s individual appointment timelines, though the average length of time to documented clinical improvement was approximately 8 weeks. Previous attempts at treating these patients’ ICI-BP were unsuccessful, leading to a variable and potentially significant delay in initiating effective treatment. None of the patients were rechallenged with their ICI, though three of the five patients continued on their respective chemotherapy regimens which were started prior to their ICI-BP. Two patients were not receiving any other treatment besides their respective ICIs.

This cohort not only fits the classical profile of a patient with BP (presenting with an average age of 74 years), but our patient population also encompassed a textbook picture of ICI-BP given that most identified as male, presented with late symptoms, and were of an older age.^1^, ^2^, ^3^, ^4^

These variations highlight the complexity of managing ICI-BP and the need for further research to optimize treatment strategies in favor of SSAs to avoid the consequences of immunosuppression.^3^ Given the variability in response duration among cases, it would be difficult to draw any conclusions on the most appropriate treatment duration. Future directions include further studies to establish more definitive guidance regarding the duration of therapy needed in this patient population. These findings regarding the length of time the patients were followed as well as the successful outcomes of dupilumab contribute to the growing body of evidence supporting dupilumab as a promising therapeutic option for ICI-BP, especially for patients where immunosuppression is not ideal.

## Conflicts of interest

None disclosed.
